# Tumor Microenvironment in Sporadic Vestibular Schwannoma: A Systematic, Narrative Review

**DOI:** 10.3390/ijms24076522

**Published:** 2023-03-30

**Authors:** Diego Cazzador, Laura Astolfi, Antonio Daloiso, Giulia Tealdo, Edi Simoni, Antonio Mazzoni, Elisabetta Zanoletti, Gino Marioni

**Affiliations:** 1 Otolaryngology Section, Department of Neuroscience DNS, University of Padova, 35100 Padova, Italy; 2 Bioacoustics Research Laboratory, Department of Neuroscience DNS, University of Padova, 35100 Padova, Italy; 3 Phoniatrics and Audiology Unit, Department of Neuroscience DNS, University of Padova, 31100 Treviso, Italy

**Keywords:** vestibular schwannoma, acoustic neuroma, tumor microenvironment, skull base, inflammation, macrophage, angiogenesis

## Abstract

Although diagnosis and treatment of vestibular schwannomas (VSs) improved in recent years, no factors have yet been identified as being capable of predicting tumor growth. Molecular rearrangements occur in neoplasms before any macroscopic morphological changes become visible, and the former are the underlying cause of disease behavior. Tumor microenvironment (TME) encompasses cellular and non-cellular elements interacting together, resulting in a complex and dynamic key of tumorigenesis, drug response, and treatment outcome. The aim of this systematic, narrative review was to assess the level of knowledge on TME implicated in the biology, behavior, and prognosis of sporadic VSs. A search (updated to November 2022) was run in Scopus, PubMed, and Web of Science electronic databases according to the PRISMA guidelines, retrieving 624 titles. After full-text evaluation and application of inclusion/exclusion criteria, 37 articles were included. VS microenvironment is determined by the interplay of a dynamic ecosystem of stromal and immune cells which produce and remodel extracellular matrix, vascular networks, and promote tumor growth. However, evidence is still conflicting. Further studies will enhance our understanding of VS biology by investigating TME-related biomarkers able to predict tumor growth and recognize immunological and molecular factors that could be potential therapeutic targets for medical treatment.

## 1. Introduction

Vestibular schwannomas (VSs) are uncommon, slow-growing benign tumors that originate from the Schwann cells lining of the axons of the eighth cranial nerve (vestibulo-cochlear nerve). Most of them arise from the vestibular nerve itself or one of its branches [1]. VSs represent about 8% of all intracranial tumors, being the most common neoplasm of the cerebellopontine angle (CPA) in adults [2]. Over the past years, VS incidence rate has been steadily increasing to about 34 VS/million/year, most probably due to easier access to improved diagnostics (e.g., Magnetic Resonance Imaging) [3]. In about 95% of cases, the VS is sporadic and unilateral, while bilateral lesions are encountered in the framework of genetic disorders such as neurofibromatosis type 2 (NF-2) or schwannomatosis [2].

The optimal management of sporadic VSs is to offer long-term cure of the disease with preservation of function, which is a realistic goal in small tumors. The choice of treatment depends on the balance between tumor and patient factors at diagnosis, such as size, growth, hearing, age, comorbidities, feasibility of sparing-function surgery and rehabilitation, and patients’ expectations. A multi-option strategy is shared with the patient, and involves an observation policy, or active treatments such as microsurgery or stereotactic radiosurgery [4]. The goal of observation is to preserve hearing and facial nerve function as long as possible in non-growing small tumors. Radiation therapy aims to stop the growth of VSs that are getting bigger. Surgery focuses on the functional outcomes (facial nerve function and hearing) and definitive cure [5,6,7]. Observational policy might expose patients to tumor growth and consequently, if surgery is then indicated, to the risks of a microsurgical resection in a bigger tumor, with potentially worse postoperative outcomes [5,6,7]. SRS aims to offer tumor stability in growing tumors, but this approach is not free of complications nor long-term functional impairments [8,9,10].

To date, the only alternative or complementary medical treatment approved for VSs is Bevacizumab, an anti-vascular endothelial growth factor (VEGF), available since 2009 [11]. It is indicated to treat growing or hearing-compromised VS in NF-2 patients; its aim is to control tumor growth over the longest period of treatment, allowing surgery to be avoided or postponed. However, Bevacizumab demonstrated less efficacy in children and its efficacy on residual VS after partial resection is low [12,13]. Therefore, there is an urgent need for novel medical treatment modalities in VSs, which preliminarily requires a better knowledge of the tumor environment.

Nowadays, there is increasing evidence that the role of the tumor microenvironment (TME) dictates tumor characteristics and evolutive features, which may play a role in the selection of the most appropriate treatment. Specifically, TME consists of cellular and non-cellular elements (e.g., the extracellular matrix components, metabolites, and cytokines) interacting together, and influencing tumor behavior [14]. The cellular compartment of TME is composed of both cells that are present in normal tissue before tumor development, and cells that are recruited from distal sites, including fibroblasts, adipocytes, immune cells, and endothelial cells [15]. Awareness of VS molecular biology and microenvironment may be of the utmost importance in investigating alternative treatments. Several attempts have been made, identifying—among others—acetylsalicylic acid and COX-2 inhibitors as potential drugs against VS growth, with debatable results [16,17,18]. However, tumor biology in sporadic VSs is still poorly understood.

The purpose of this systematic, narrative review was to assess the level of current knowledge on the role of TME in VS. A high awareness of the TME in VS would be useful to: (i) investigate biomarkers predicting tumor evolution, and (ii) recognize immunological and molecular factors that could be potential targets for medical treatment in the near future.

## 2. Materials and Methods

### 2.1. Protocol Registration

The protocol of this systematic review and meta-analysis was registered on PROSPERO, an international database of prospectively registered systematic reviews in health and social care (Center for Reviews and Dissemination, University of York, York, UK), in November 2022 (registry number CRD42022366609).

### 2.2. Search Strategy

A systematic literature review was conducted according to the Preferred Reporting Items for Systematic Reviews and Meta-Analyses (PRISMA) recommendations [19]. The electronic databases Scopus, Pubmed, and Web of Science were searched from database inception to 15 November 2022. A combination of MeSH terms and free-text words were utilized to search for: “vestibular schwannoma”; “acoustic neuroma”; “microenvironment”; “immunohistochemistry”; “growth factor” (Supplementary Materials). The reference lists of all the included articles were thoroughly screened to find other relevant articles. References were exported to Zotero bibliography manager (v6.0.10, Center for History and New Media, George Mason University, Fairfax, VA, USA). After duplicates removal, two reviewers (A.D. and D.C.) independently screened all titles and abstracts and then evaluated the full texts of the eligible articles based on the inclusion criteria. Any disagreement between the reviewers involved in the literature search was resolved through discussion with all authors to reach a consensus.

### 2.3. Selection Criteria

Studies were deemed eligible when the following inclusion criteria were met: (i) confirmed pathological diagnosis of sporadic VS; (ii) tissue specimen analysis performed through immunohistochemistry (IHC) or molecular methods; (iii) tumors primarily treated with surgery. Exclusion criteria were as follows: (i) retrospective series with less than 10 cases; (ii) NF-2 patients; (iii) previously irradiated tumors; (iv) lack of relevant data; (v) non-original studies (i.e., reviews, recommendations, letters, editorials, or book chapters); (vi) animal model studies, (vii) non-English studies. The papers were thoroughly screened for duplicates.

### 2.4. Data Extraction and Quality Assessment

Extracted data were collected in an electronic database including first author, year of publication, country of origin, study design, sample size, number of patients included, mean age of the patients, sex ratio, mean tumor size, investigated biomarkers, methods applied for biomarkers detection, study aim, key findings. The quality of the studies eligible for inclusion was categorized as Poor, Fair, and Good, in agreement with the National Institutes of Health quality assessment tool for Observational Cohorts and Cross-Sectional Studies (https://www.nhlbi.nih.gov/health-topics/study-quality-assessment-tools, accessed on 15 November 2022) [20]. Two reviewers (A.D. and D.C.) independently evaluated the papers, and any disagreement was resolved by consensus.

## 3. Results and Discussion

### 3.1. Search Results and Quality Assessment

**Figure 1:**
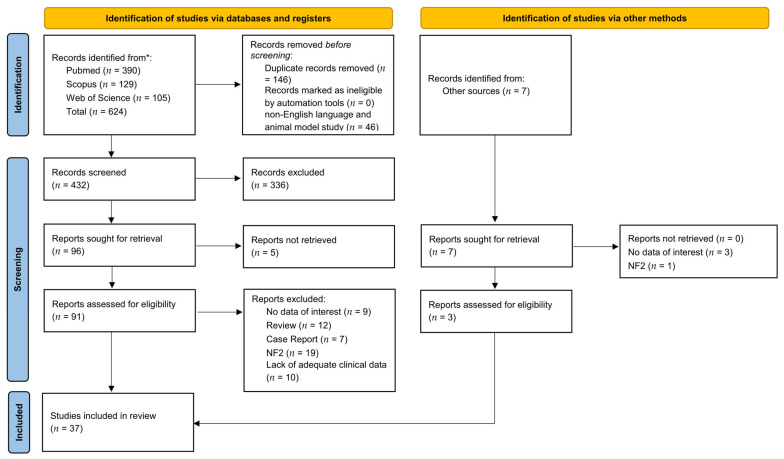
PRISMA diagram summarizing Electronic Database Search and Inclusion/Exclusion process of the review. * date of last search 15 November 2022.

A total of 624 titles were collected from our literature search. After duplicates removal and exclusion of 336 records due to coherence with the inclusion/exclusion criteria, 103 articles relevant to the topic were examined. Five records were unavailable for retrieving. Ninety-four articles were assessed for eligibility, and, in the end, 37 were included in the review [21,22,23,24,25,26,27,28,29,30,31,32,33,34,35,36,37,38,39,40,41,42,43,44,45,46,47,48,49,50,51,52,53,54,55,56,57]. A detailed flowchart of the search process is shown in Figure 1. Available data in the included manuscripts were inadequate to perform a quantitative analysis.

In accordance with the National Institutes of Health quality assessment tool for Observational Cohorts and Cross-Sectional Studies [20], 12 studies were deemed of Good quality (32.5%), 16 Fair (43.2%), and only 9 (24.3%) were classified as Poor, due to the lack in reporting information on the series’ features (Table S1).

### 3.2. Included Studies’ Characteristics

All 37 studies included in the qualitative analysis had an observational retrospective design, and were ex vivo tissue investigations based on histopathological analysis of surgical specimens [21,22,23,24,25,26,27,28,29,30,31,32,33,34,35,36,37,38,39,40,41,42,43,44,45,46,47,48,49,50,51,52,53,54,55,56,57]. Studies were published from 1990 to 2022. The median number of patients per study was 32 (range 10–923).

Major findings of the retrieved articles are discussed in dedicated paragraphs, and data on patients’ demographics, study design, tumor characteristics, and relevant conclusions of each article included, are reported in Table 1, Table 2, Table 3, Table 4, Table 5 and Table 6.

The current knowledge and debate on TME in VS presented in the eligible articles focused on: (i) angiogenesis; (ii) immune cells infiltration; (iii) molecular regulators; (iv) growth factors; (v) matrix metalloproteinases; and (vi) hormone receptors.

### 3.3. Angiogenesis

Tumor cells secrete several factors leading to the transformation of a previously anti-tumorigenic milieu into a pro-tumorigenic one [58]. Tumors interact with numerous stromal cells, including local and infiltrating fibroblasts, macrophages, mesenchymal stem cells, endothelial cells, pericytes, secreted factors, and the extracellular matrix within the TME [59]. Angiogenesis is a major prerequisite for the proliferation and progression of several neoplasms [60]. Judah Folkman, considered the “father of angiogenesis”, proposed the first model of tumor angiogenesis according to which tumor cells are able to sense their increasing distance from existing vasculature and, in response, release angiogenic signals [61]. Currently, tumor angiogenesis is considered the result of an imbalance between pro- and anti-angiogenic factors produced by tumor and physiological cells [62].

Among the various pro-angiogenic growth factors, the vascular endothelial growth factor (VEGF) is a major regulator of angiogenesis [63]. Previous studies analyzed different types of brain tumors for VEGF expression [64,65]. Furthermore, VEGF has been reported to be a survival factor for Schwann cells [66]. This result suggests a crosstalk between tumor cells and vessels in a paracrine-stimulating manner.

Even though VS are generally slow-growing tumors, a functional vascular system still remains important for tumor growth [39]. In 1996, Matsugana et al. [48] hypothesized that VSs might shrink spontaneously or remain silent for a long time if there was poor angiogenesis. Whereas they might grow or regrow rapidly in the presence of significant angiogenesis [48].

Investigating the angiogenic effect of VS on the adjacent vestibulocochlear nerve, an increased vascularization was found showing delicate blood vessels, susceptible to mechanical stress [48]. These data suggested that one of the most plausible explanations for hearing loss in VS was edema of the cochlear nerve due to circulatory disturbance and that postoperative hearing loss was due to surgical injury of the vessels running between tumor and nerve [48].

In VS, a relationship was found between micro-vessel density (MVD), tumor size, and growth rate with an ultrastructural approach [41,67], while other studies demonstrated tumor expression of VEGF with immunohistochemical methods [68,69]. Brieger et al. [26] analyzed angiogenesis in 34 VS specimens, concluding that VEGF, VEGF-R1, VEGF-R2, and TGF-β1 were unexpressed in tumor tissue, with the exception of one case. MVD investigated through CD31 staining revealed low expression in the specimens [26]. On the other hand, other studies on MVD in VS showed divergent results, with MVD being positively correlated with tumor size and growth [29,44]. Furthermore, these studies evidenced an association between angiogenic factors and MVD with CD68 positive cells and macrophage density in tumor tissue [29,44].

Cayè-Thomasen et al. [28] found that the high-affinity receptor VEGFR-1 was expressed in VS, and also that the tumor homogenate concentration of both VEGF and VEGFR-1 correlated positively with tumor growth rate but not with tumor size. These findings suggested that VEGF might also play a role in benign neoplasms’ growth. Accordingly, VS growth could be associated with high levels of VEGF in both blood and cerebrospinal fluid, therefore providing a growth indicator. Consequently, VEGF measurement may become a valuable tool for therapeutic approach choice [28].

Koutsimpelas et al. [38] tried to clarify the role of VEGF and basic fibroblast growth factor (bFGF) in VS vascularization and growth. They found a significant association between VEGF, bFGF, MVD, and tumor growth index. Furthermore, bFGF was associated with tumor volume in VS. They hypothesized that accelerated growth could be related to higher bFGF levels, resulting in larger tumors.

It was found that recurrent VS and tumors treated with radiosurgery prior to operating had a considerably elevated VEGF expression in comparison to the primary tumors, probably because of surgical trauma or an intrinsic propensity [39]. It was suggested that these tumors express high levels of VEGF per se, and the increased VEGF levels might have a role in protecting the endothelial and/or tumor cells during the radiation. Consequently, the failure of radiosurgery to control VS tumors might be due to high VEGF levels [39]. It was hypothesized that recurrent VS might be targeted using antiangiogenic active compounds. Furthermore, if it was proven that VEGF mediated VS protection through reduction of radiation sensitivity, then a combination of radiotherapy and anti-VEGF treatment might improve therapeutic outcome [39].

**Table 1:** Studies examining the role of angiogenetic factors in sporadic vestibular schwannomas.

Author	Year	Country	Study Design	Cases (*n*)	Mean Age ± SD (Range)	Sex (M/F)	Tumor Size, Mean ± SD (Range)	Method of Marker Detection	Marker Studied	Key Findings
Brieger et al. [26]	2003	Germany	Ex vivo tissue study	34	49 (19–72)	20/14	16.5 mm (5–36)	IHC	VEGF VEGF-R1 VEGF-R2 TGF-β1 CD31 CD68	- VSs did not express or expressed very low levels of pro-angiogenic growth factors - Tumor angiogenesis did not seem a relevant mechanism of VS growth
Cayè-Thomasen et al. [28]	2005	Denmark	Ex vivo tissue study	27	53 (35–61)	11/16	1.431 cm^2^ (0.198–4.589)	ELISA	VEGF VEGF-R1	- The concentration of VEGF and VEGFR-1 correlated with tumor growth rate but not with tumor size
Koutsimpelas et al. [38]	2007	Germany	Ex vivo tissue study	17	51.5 ± 12.2 (28–71)	11/6	424 ± 658 mm^3^ (36–2556)	IHC qPCR	VEGF bFGF	- The bFGF and VEGF mRNA expression and their protein expression correlated with tumor volume, tumor growth index, and MVD
Koutsimpelas et al. [39]	2012	Germany	Ex vivo tissue study	182	52 ± 10.6 (18–78)	79/ 103	2.404 ± 2.329 mm^3^ (24–37.679)	Tissue microarray IHC	VEGF VEGFR-1 VEGFR-2 NP1	- A relevant role of the VEGF pathway in VS growth and therapy outcome was suggested - Targeting antiangiogenic pathway might be useful in sporadic VS patients
Marioni et al. [46]	2019	Italy	Ex vivo tissue study	71	52.8 ± 13.0	37/34	10 intra-meatal; 21 small (<1 cm); 27 medium-sized (1–2.5 cm); 13 large (>2.5 cm)	IHC	Endoglin (CD105)	- Positive correlation between preoperative tumor size, and (i) vessel cross-sectional area, (ii) CD105-assessed vessel density No significant correlation between tumor growth rate and vessel cross-sectional area or vessel density
Matsunaga et al. [48]	1996	Japan	Ex vivo tissue study	29	51.38 ± 12.18 (8–66)	13/16	19.03 ± 10.32 mm (5–40)	IHC	Blood vessels	- New blood vessels were more fragile than those in the normal VIII cranial nerve - SNHL in VS patients may be associated with damage to fragile blood vessels. - Surgical injury to fragile vessels may cause postoperative hearing loss
Xia et al. [57]	2020	China	Ex vivo tissue study	38	NR	NR	31.95 ± 1.74 (NR)	IHC	MMP-14 VEGF	- MMP-14 and VEGF expressions were significantly higher in the Antoni B than in the Antoni A area - Upregulated MMP-14 may degrade loose collagen in the Antoni B area determining cystic formation - MMP-14 can enhance VEGF activity, which may cause plasma ultrafiltrate extravasation, cystic expansion, and intra-tumoral hemorrhage. - MMP-14 inhibition may be a therapeutic approach for cystic VSs

F: female; M: male; MVD: microvessel density; *n*: number of cases; SD: standard deviation; NR: not reported; VS: vestibular schwannoma.

Marioni et al. [46] first investigated neoangiogenesis in sporadic VS using CD105 staining. CD105 resulted in a useful marker to identify proliferating endothelium involved in tumor angiogenesis [70]. They found a significant positive correlation between tumor size at the time of surgery and (i) vessel cross-sectional area as well as (ii) CD105-assessed vessel density.

Quite recently, the different expression of matrix metalloproteinases (MMPs) and VEGF in the Antoni A and B areas was investigated [57]. The authors evidenced a significantly higher MMP-14 and VEGF expression in the Antoni B than in the Antoni A areas, and concluded that the upregulation of MMP-14 induced the degradation of loose collagen in the Antoni B area, thus contributing to cystic formation. Moreover, the overexpression of VEGF (which is also enhanced by MMP-14) increased the permeability of tumor vessels and caused cyst fluid leakage from the fragile neo-vasculature in the Antoni B area, which determined cystic expansion and intra-tumoral hemorrhage.

Based on these results, angiogenesis seems to have an impact on TME, for instance by influencing tumor growth index and prognosis [29,39,48]. Overall, VS progression may be decreased by modulating the characteristics of TME through the inhibition of cytokines and growth factors (e.g., VEGF, bFGF, M-CSF, IL-34, etc.) [28,29,38]. In vivo VS models are necessary to clarify the biological mechanisms that are involved in the associations of TME mutations and VS behavior.

### 3.4. Immune Cells Infiltrates

Immune system functions are involved in several processes in tumor tissues, from immunosurveillance to immune escape, when the tumor overcomes immunological control, leading to tumor growth [71]. Immune cell infiltration in the VS tissue microenvironment has long been observed, in particular for T and B lymphocytes and macrophages; however, their definitive role in tumorigenesis, growth, or patients’ symptoms such as hearing deterioration remains to be established.

One of the first studies examining the inflammatory phenotype in VSs revealed a positive correlation between tumor tissue inflammation (investigated through CD45 staining) and the duration of clinical symptoms [41]. Having observed that the degree of tumor tissue inflammation increased with time, it was postulated that inflammation could be a degenerative process in TME [41].

The relationship between angiogenesis and immune cells infiltration in VS received increased attention when the role of tumor-associated macrophages (TAMs) was investigated. TAMs came into play in TME of solid tumors with a prominent role in the regulation of tumor growth, invasion, and angiogenesis [14,72]. Specifically, among the heterogeneous population of TAMs, M2-type macrophages—identified through the cell surface marker CD68—seem to have tumor-promoting features and seem to be associated with poor outcomes in different gastro-intestinal malignancies [73]. de Vries et al. [29] found a significant MVD increase in VSs with a high number of CD68 positive cells (macrophage markers). More recently, the same research group investigated the role in VS of the macrophage colony stimulating factor M-CSF, a cytokine that regulates macrophage recruitment, proliferation, and differentiation, polarizing macrophages towards a pro-tumoral M2-like phenotype in TME. de Vries et al. [30] reported a significantly higher expression of M-CSF in fast-growing VSs, as well as in cystic tumors. Analogously, CD163 expression was significantly elevated in tumors with strong M-CSF staining [30]. This evidence shed light on the potential role of M-CSF as a target for specific inhibitors.

The existing association between sporadic VS vascularity and TAM infiltration was further corroborated by Lewis et al. [44]. Higher MVD and fibrinogen density (marker of vessel permeability) were found in high-TAM-density tumor regions. Furthermore, a spatial correlation of areas of high TAM density and (i) VEGF and (ii) VEGFR-1 expression was observed. Thus, it is likely that VEGF/VEGFR-1 signaling promotes chemo-attraction of VEGFR-1-expressing TAMs in the TME. In this context, treatment directed against the VEGF pathway might reduce both TAM effect and angiogenesis.

Many attempts have been made to identify potential immune cell factors influencing the growth of sporadic VS. A series of 67 VSs was evaluated for the leukocyte antigen CD45 and for CD68 positive cells, revealing a positive correlation among their expression, tumor size, and tumor growth index [29]. M2 macrophages involvement was analyzed by the same researchers [30] in two groups of 10 slow- and 10 fast-growing VSs. The expression of CD163 was found to be significantly higher in fast-growing tumors, highlighting the potential involvement of M2 TAMs in growth progression. In this sense, Leisz et al. [43] investigated the role of TME in association with VS volume and growth rate. They detected increased macrophage markers expression (CD163 and CD68) in volumetrically larger tumors, an observation that was confirmed by Gonçalves et al. [34] considering 923 VSs. In the Leisz series, growth rate was determined in a subset of 74 patients, revealing significantly higher expression of CD68 in fast-growing tumors (> 1 cm^3^/year) [43]. These findings were confirmed on a subset of randomly selected growing tumors, where fast tumor growth was associated with higher levels of CD163 and CD68 positive cells. Similar observations were reported by Lewis et al. [44] demonstrating higher TAM density in rapidly growing VSs. Contrarily to the previous results, Gonçalves et al. [34] proved an inverse relationship between tumor growth rate and tissue inflammation. The authors elaborated an inflammatory score encompassing CD3, CD8, CD68, and CD163 expressions. Surprisingly, higher inflammatory score levels were independently associated with slower volumetric tumor growth. Thus, for the first time, growth rate in VS and its inflammatory microenvironment were found to be inversely correlated, demonstrating the need for further detailed studies on the role of immune response in VS.

Two investigations [23,53] tried to define the immune signature of VSs that underwent sub-total microsurgical resection and observed the evolution of tumor residuals. Amit et al. [23] found a different tumor-immune environment between the collected specimens of rapidly progressing and slowly or not-progressing residuals, in both the innate and adaptive immune compartments. Particularly, rapidly progressing tumors presented a significant enrichment of CD68+ macrophages, CD4+ and CD8+ T lymphocytes, and CD20+ B cells, whereas a lower density of dendritic cells positive for CD1a. To further explore these findings, a transcriptomic analysis was conducted revealing differences in gene expression and pathways dysregulation. Early progressing residuals revealed immune signaling downregulation and enriched cellular senescence pathways linked to viral infection (e.g., NF-kB pathway); thus, leading the authors to hypothesize a possible viral etiology in fast progressing VSs.

On a cohort of operated VSs, a significantly increased TAM density in tumors that progressed after sub-total resection was confirmed [53]. Remarkably, this research group reported contrasting results to what had previously been observed by other teams. Significantly higher CD163 positive cell density and increased M2 index were encountered in VSs that remained stable after sub-total resection, than in progressive tumors. Given these results, the authors concluded that TAM phenotype in VS might be far more elaborate than ordinary M1/M2 polarization. High density of M2-like macrophages in stable tumors could reasonably be considered as the result of the anti-tumor host response.

**Table 2:** Studies examining the role of immune cell populations in sporadic vestibular schwannomas.

Author	Year	Country	Study Design	Cases (*n*)	Mean Age ± SD (Range)	Sex (M/F)	Tumor Size, Mean ± SD (Range)	Method of Marker Detection	Marker Studied	Key Findings
Amit et al. [23]	2022	USA	Ex vivo tissue study	17	51.3 ± 11.22	6/11	NR	CD4/CD8 CD20 CD68 CD1A	IF	- Multiple pathways are dysregulated in rapidly progressing forms of sporadic VS - The drivers of VS progression seemed to be external rather than internal genetic perturbations
De Vries et al. [29]	2012	Netherlands	Ex vivo tissue study	67	49.04 ± 14.06 (15–72)	26/41	24.03 ± 11.52 mm (5–50)	Histone H3 Ki-67 CD31 CD45 CD68 Hemosiderin	IHC	- Intratumoral hemosiderin, MVD and inflammation were positively correlated with tumor size and the tumor growth index - MVD was significantly higher in CD68+ tumors
De Vries et al. [30]	2019	Netherlands	Ex vivo tissue study	20	56.75 (39–81)	4/16	13 ± 7.44 mL	M-CSF IL-34	IHC	- M-CSF was related to macrophage activity and tumor progression. - IL-34 role remained unclear
Gonçalves et al. [34]	2021	Germany	Ex vivo tissue study	923	NR	NR	4.73 cm^3^ (0.04–52.14)	CD3 CD8 CD68 CD163	IHC	- Inflammatory cell infiltration increased in larger VSs - Inflammatory cell infiltration was associated with slower percentage of volumetric growth
Labit-Bouvier et al. [41]	2000	France	Ex vivo tissue study	69	53 (median) (20–77)	37/32	18 mm (median) (6–50)	CD34 CD45 ER, PR	IHC	- No clinical parameters were proved to be predictive of growth - Tumor size was significantly related to vessels’ number - Significant association between clinical growth index and total vessels’ number - No relationship between CD34 and symptoms’ duration - The degree of inflammation was significantly related to symptoms’ duration - No estrogen receptors were detected; only a few tumors expressed progesterone receptors without any significant clinical value
Leisz et al. [43]	2022	Germany	Ex vivo tissue study	74	53 (28–77)	32/42	2.44 cm^3^ (0.1–18.8)	Ki-67 COX2 VEGF M-CSF GM-CSF CD163 CD68	RT-PCR	- Negative correlation of the Ki-67, COX2 and VEGF on tumor volume - With a higher volume of VS, the expression of CD68, CD163 and GM-CSF increased significantly
Lewis et al. [44]	2021	UK	Ex vivo tissue study	17	49.4 (median) (41.3–55-8)	7/10	2.51 cm^3^ (median) (1.56–5-91)	Iba+ CD31 Fibrinogen TAM VEGF VEGFR-1 Ki-67	IHC IF DCE-MRI	- Transfer constant, tissue extravascular-extracellular space, and tumoral free water content increased with increasing VS size and pretreatment growth rate - Close association between vascularity and Iba1+ macrophage density
Perry et al. [53]	2020	USA	Ex vivo tissue study	46	57 (median) (25–83)	21/25	Stable disease 3.3 cm (1.8–5.2) Tumor progression 2.9 cm (1.8–5.0) (median)	CD68 CD163 PD-L1	IHC	- Significantly increased levels of M1, CD163+ TAMs in association with VS that progressed after subtotal resection - Progressive VSs were characterized by increased PD-L1, because of a mechanism of immune evasion resulting in TAM deactivation, tumor growth and infiltration of anti-tumor immune cells - Targeting PD-1/PD-L1 could have a role in disease control after subtotal resection

ceMRI: contrast-enhanced magnetic resonance imaging; F: female; M: male; MVD: microvessel density; *n*: number of cases; NR: not reported; SD: standard deviation; TAM: tumor-associated macrophage; VS: vestibular schwannoma.

### 3.5. Molecular Regulators

The VS microenvironment is finely modulated by the interplay of several cellular pathways leading to dysregulation of the natural physiological balance between programmed cell death and cell proliferation. The primary molecular events inducing VS, in both sporadic and NF-2 related VS, are the occurrence of Nf2 gene mutations on chromosome 22 and subsequent loss of the tumor suppressor protein Merlin. Merlin is a protein of FERM (4.1 protein/Ezrin/radixin/moesin) that plays a role in cell attachment, cell motility, membrane receptor availability, signal transduction, and then in cell proliferation. Merlin’s antitumor activity is anti-proliferative. Its overexpression induces cell cycle arrest in G1 phase, decreasing P21 expression and inhibiting the cell cycle regulators as cyclins [35]. The inactivation of Merlin detected in VS results from the phosphorylation of serine 518 that is exerted by the activity of P21 activated kinase (PAK), which in turn is activated by ras-related C3 botulinum toxin substrate 1 (Rac1) [74,75]. Rac1 drives the progression of the cell cycle, from G1 to the synthesis phase, promoting the cyclin D1 expression. Among the cyclins in VS patients, it has been demonstrated that both cyclin D1 and cyclin D3 play a role in cell proliferation [35,42,52]. There is no consistency in the evidence reported about a direct correlation between cyclin D1 and cyclin D3 expression and the proliferation index Ki-67 [35,42,52]. Ki-67 is a nuclear protein expressed during cell replication. This evidence suggests that VS event undergoes different mechanisms that regulate cell proliferation [35,52]. Cyclin D1 activity is expressed early in the G1 phase; it binds to cyclin-dependent kinases CDK4 or CDK6, that once activated phosphorylate the target protein retinoblastoma Rb. The latter induce E2F transcription factors to activate the expression of S-phase genes and thus the progression of the cell cycle [42]. Based on the foregoing, a possible predictor of VS aggression may be the deregulation of a factor involved in cyclin D-CDK binding during the transition from G1 to S as P27 protein. The P27 together with P21e P57 are included in the Cip/Kip family; they influence the cyclin-CDK complexes formation during the progression from G1- to S-phase. In fact, the p27 and p21 are cyclin-dependent kinase inhibitors and the p27 expression was observed inversely related to the Ki-67 proliferation index [55].

Another gene involved in cell cycle progression is the p53 transcription factor. This tumor suppressor acts at the control point of the cell cycle, where, in response to specific cellular stress stimuli, p53 binds to a specific tract of DNA to induce the arrest in G1 or apoptosis [55]. Its involvement in VS is still unclear. Several authors have reported that its dysfunction is given by the loss of heterozygosity in the region of the first gene intron, others attribute it to a phosphorylation (resulting age-related), others have not found a correlation with tumor aggressiveness in terms of p53 expression [21,55]. A homologous gene of p53 is P73. It is very similar (60% homology) in terms of domain structure, conformation and functions. The P73 can be translated into two different isoforms, one full length and active and one truncated and inactive. The former acts as an onco-suppressor like P53, whereas the latter isoform has an oncogenic action. The latter, by blocking the transactivation of p53 and the active form of P73, hinders its apoptotic activity. On the other hand, both p53 and the active form of P73 induce expression of the truncated form of P73, producing a complex feedback loop [21]. In VS, the expression of P73 was demonstrated in 40% of the samples analyzed, but which isoform corresponded was not defined. In other epigenetic studies in schwannomas, an aberrant DNA methylation of various genes including P73 was reported, explaining the deregulation of tumor suppressors [21].

**Table 3:** Studies examining the role of molecular regulators in sporadic vestibular schwannomas.

Author	Year	Country	Study Design	Cases (*n*)	Mean Age ± SD (Range)	Sex (M/F)	Tumor Size, Mean ± SD (Range)	Method of Marker Detection	Marker Studied	Key Findings
Ahmad et al. [21]	2009	Spain	Ex vivo tissue study	34	49.5 (25–72)	19/15	17 mm (3–40)	IHC, cell culture WB, IF, Flow citometry	p73	- p73 can alter the cell-cycle distribution and induce cell death - p73 may have a role in the clinical setting by either its direct administration via gene therapy or by using it in combination with stereotactic radiosurgery
Breun et al. [24]	2018	Germany	Ex vivo tissue study	30	51 (NR)	12/18	10 small tumors (T3A or smaller) 20 large tumors (T3B of larger)	IHC WB RT-qPCR	CXCR4 CXCL12	- CXCR4 mRNA was overexpressed in VS in comparison with normal VIII cranial nerves - CXCR4 may be a prognostic marker - CXCR4 inhibition has potential for a systemic approach to VS
Jabbour et al. [35]	2016	Australia	Ex vivo tissue study	180	54 ± 13.9 (14–81)	96/84	NR	IHC	cyclin D1 cyclin D3 Ki-67	- Loss of functional merlin is common in both sporadic and NF2 VSs - This loss contributes to cell cycle pathway deregulation, with loss of Rac signaling inhibition - This may lead to cyclin D protein overexpression promoting VS growth - Overexpression of cyclins D1 and D3 is a common feature in VS
Lassaletta et al. [42]	2011	Spain	Ex vivo tissue study	64	49 (16–78)	27/37	23 mm (5–55)	IHC	Cyclin D1	- Cyclin D1 expression is associated to facial outcome after VS surgery - The prognostic value of cyclin D1 expression was independent of tumor size and facial nerve stimulation after surgery
Martini et al. [47]	2017	Italy	Ex vivo tissue study	36	51.8 ± 12.4 (NR)	17/19	4 intra-meatal; 8 small-sized (<1 cm); 19 medium-sized (1–2.5 cm); 5 large-sized (>2.5 cm)	IHC ceMRI	YAP TAZ AREG	- Expression of TAZ correlated significantly with VS volume measured on ceMRI
Mawrin et al. [49]	2002	Germany	Ex vivo tissue study	14	58.36 ± 5.35 (52–73)	7/7	NR	IHC	Fas-Fas-L Bcl-2 Bax MIB-1	- No significant correlations between different labeling indices - VS expressing Bax tended to show a higher proliferation rate - Fas-L was present in most VS but due to the lack of Fas expression, apoptosis in VS did not seem to be mediated via the Fas-Fas-L system
Neff et al. [52]	2006	USA	Ex vivo tissue study	15	NR	NR	NR	IHC	Cyclin D1 Cyclin D3	- Cyclin D1 protein did not play a prominent role in promoting cell cycle progression - Cyclin D3 expression was found in nearly half of the VSs, suggesting that it could have a growth-promoting role
Seol et al. [55]	2005	Korea	Ex vivo tissue study	12	NR	NR	NR	IHC	p53 Bax Bcl-2 Fas Fas-L Caspase-3 p27 p21	- The loss of p27 in VS may explain the higher proliferative potential of aggressive VS versus usual VS: p27 may be a predictor of VS aggressiveness - The expressions of other apoptosis associated proteins were not significantly different

ceMRI: contrast-enhanced magnetic resonance imaging; F: female; M: male; *n*: number of cases; NR: not reported; SD: standard deviation; VS: vestibular schwannoma.

The loss of Merlin function in the VS occurrence could activate other two main signaling pathways, Ras/Raf/MEK pathway and PI3K/Akt/mTor pathway, which, by inducing the inhibition of apoptosis, lead to increased cell survival and proliferation [24]. The tumorigenesis is linked to the activation of IP3/Akt and MAP signaling, downstream of the activation of Chemokine-4 receptor (CXCR4) [24]. In a recent study, it was reported that CXCR4 was similarly overexpressed in both sporadic and NF2-associated VS, with no significant association with the size or extent of the tumor. Interestingly, a positive correlation has been seen between its expression and the level of hearing impairment, from which the authors suggested an association with the increasing invasiveness of the tumor [24]. The CXCR4 is a G protein coupled receptor known for its activity during embryogenesis and used in various therapies, including cancer. Its only ligand is CXCL12; their binding leads to the activation of IP3/Akt and MAP signaling, phosphorylation ERK1/2, and calcium release, resulting in increased tumor invasiveness and proliferation [24].

Based on the above considerations, it appears clear that TME promotes its development as a consequence of the deregulation of cell proliferation and/or apoptosis. Intrinsic apoptosis is activated by two main pathways: the first involves activation of cell surface receptors (such as Fas/Fas-L), while the second involves release of mitochondrial cytochrome C into the cytosol. Available evidence has made it possible to exclude the first route. As for the second, a balance between the expression of the apoptosis inhibitor Bcl-2 and the pro-apoptotic factor Bax was detected [55]. It should be noted that Bcl-2 can exert its anti-apoptotic action in two ways, on the one hand interacting with Fas induces the lack of Fas expression, on the other forming heterodimers with Bax inhibits its pro-apoptotic effect [49,55]. It has been suggested that in VS a kind of balance exists between proliferation and cell death maintained by the pro- and anti-apoptotic factor expression and the related increase of the Ki-67 proliferation index [49,55].

The Merlin’s loss of function also influences other pathways, like the Hippo pathway and the VEGF-mediated signaling pathway. The first downstream effectors of the Hippo signaling pathway are Yes-associated protein (YAP) and its paralog transcriptional co-activator with PDZ-binding motif (TAZ) [47,76]. Translocation of TAZ and YAP from the cytoplasm to the nucleus leads to activation of various transcription factors including p73, T-box transcription 5, SMAD family proteins and several members of the TEA domain (TEAD) family. The TEAD family is known to be the most common in the regulation of gene expression related to cell proliferation and apoptosis [47,76,77]. Another target gene of YAP and TAZ is Amphiregulin (AREG), a member of the epidermal growth factor family that has been identified as a schwannoma-derived growth factor. In a recent study, a significant positive correlation between VS volume and the expression of TAZ, not of YAP or AREG, was found. For this reason, the authors assumed that TAZ likely promoted the transcription of other target genes [47]. By an analysis of gene expression profiling, it was demonstrated that the increased nuclear expression of YAP was positively correlated with the Ki-67 proliferation index in VS [77].

### 3.6. Growth Factors

In VS, the TME that then initiates progression of the tumor is given by the cascade of molecular death or cell proliferation pathways, which implement downstream of Merlin dysregulation. Regarding the growth and aggressiveness of cancer, deregulation of the expression of growth factors and their receptors plays a fundamental role. Intriguingly, a positive correlation between the proliferation index and the gene expression of different neurotrophic factor was detected in VS microenvironment [40,56]. The brain-derived neurotrophic factor (BDNF), co-receptor Ret and the transforming growth factor (TGF)-β1 were found overexpressed in VS, but only BDNF was positively correlated to the Ki-67 proliferation index [40]. The BDNF and NT-3 are involved in the myelination of axons and their correspondent receptors are p75NTR, tyrosine kinase (Trk)-B and Trk-C. It is not yet clear how BDNF and p75NTR and Trk-B pathways are involved in VS, but it was hypothesized that the binding of BDNF-p75NTR leads to myelination, which is reflected in the loss of axonal contact in benign schwannomas. In the absence of p75NTR, myelination is inhibited due to Trk-B expression, which blocks Trk-C signaling leading to suppression of NT-3 mediated myelination [40]. The TGF-β1 is activated by two receptors, the threonine/kinase transmembrane receptor 1(TGF-βR1) and TGF-βR2 [31,45,56]. The binding to the first receptor has a proliferative activity, on the contrary, the binding to the type 2 receptor leads to an anti-proliferative action [45,56]. It has been suggested that TGF-β1 may also be indirectly involved in tumor progression by promoting angiogenesis mediated by VEGF overexpression [56]. VEGF induces angiogenesis through the proliferation and migration of endothelial cells. This is activated by VEGFR-1 and VEGFR-2 receptors. Their binding leads to the extravasation of plasma proteins from the tumor vessels, which leads to the formation of a temporary extra-vascular matrix that results in the formation of new blood vessels due to migration and proliferation of endothelial cells [56]. Note that angiogenic activity of the VEGF pathway has also been shown to be related to loss of Merlin function [47,56,76]. Expression of TGF-β1 was also analyzed in synergy with the growth factor glial-cell-derived growth factor (GDNF) [31]. The two factors belong to the same superfamily of growth factors and are involved in the neurotrophic action. In particular, their co-expression has been reported in most of the VS samples analyzed, reinforcing the theory that they act in concert to stimulate neurotropicity in sympathetic neurons [31].

Among other growth factors involved in VS together with the loss of Merlin function, neuroregulin 1 and Platelet Derived Growth Factor (PDGF) were studied [22,78,79]. The binding of neuregulin 1 to its receptor ERBB2 induces the mitogenic action of Schwann cells followed by a cascade of phosphorylation involving the Pl3k and MAPK system [18,19,20], leading to the mitogenic effect. Regarding hearing loss following tumor development in patients with VS, it has been shown that while PDGF expression is positively related to age and hearing loss; it is not related to tumor size [22,33]. At the same time, the Fibroblast Growth Factor 2 (FGF2) appears to have a protective effect against hearing loss despite its expression being directly related to the size of the tumor [33]. Finally, the expression of ERBB2 having proliferative effects is not related to hearing loss [33,78,79]. In conclusion, all this information gathered about the VS microenvironment could help to obtain customized therapeutic protocols dosing inhibitory molecules with stimulants, allowing the tumor to be reduced and hearing preserved [33].

**Table 4:** Studies examining the role of growth factors in sporadic vestibular schwannomas.

Author	Year	Country	Study Design	Cases (*n*)	Mean Age ± SD (Range)	Sex (M/F)	Tumor Size, Mean ± SD (Range)	Method of Marker Detection	Marker Studied	Key Findings
Altuna et al. [22]	2011	Spain	Ex vivo tissue study	34	49.5 (25–72)	19/15	17 mm (3–40)	IHC, cell culture and WB, IF, Colony Formation Assay	PDGF-R c-Kit	- c-Kit and PDGFR-β were highly expressed in a significant proportion of VS - Gleevec can downregulate phosphorylation of both PDGFR-β and c-Kit in HEI-193 at clinically relevant doses
Diensthuber et al. [31]	2004	Germany	Ex vivo tissue study	22	55.3 ± 11.8 (27–77)	13/9	17 ± 6.8 mm (7–30)	IHC	TGF-β1 Glial Cell Line-Derived Neurotrophic Factor Ki-67 TβR II, GFRα-1 and Ret	- Expression of TGF-β1 and glial cell line–derived neurotrophic factor may suggest their biological role in VS - Trophic TGF-β1/glial cell line-derived neurotrophic factor synergism seemed possible and was underscored by co-expression of both neurotrophic factors and their receptors
Dilwali et al. [33]	2013	USA	Ex vivo tissue study	16 gh 19 ph	NR	NR	NR	Cytokine Array ELISA	FGF2	- FGF2 is a potential tumor-secreted mediator of hearing protection in VS
Kramer et al. [40]	2010	Germany	Ex vivo tissue study	18	54 (20–77)	NR	NR	RT-qPCR IHC	BDNF, GDNF TGF-β1/β2 Ki-67	- BDNF expression was observed in the VS, whereas gene expression of artemin and GDNF was upregulated in peripheral nerves - The association between Ki-67 level index and BDNF, TGF-b1 and Ret was significant in the VS - Coherence was found between BDNF expression and proliferative activity in VS
Löttrich et al. [45]	2007	Germany	Ex vivo tissue study	40	NR	NR	NR	IHC qRT-PCR	TGF-β R1 TGF-β R2	- TGF-β/TGF-β R1 and -R2 system were present in VS
Taurone et al. [56]	2015	Italy	Ex vivo tissue study	10	NR (45–69)	6/4	NR	IHC	TGF-β1 IL-1β IL-6 TNF-α ICAM-1 VEGF	- Neoplastic Schwann cells produce pro-inflammatory cytokines that may act in an autocrine manner, stimulating cellular proliferation - The increased expression of VEGF suggested that this factor might induce neoplastic growth via angiogenesis. - Inflammation might promote angiogenesis and consequently contribute to tumor progression - VEGF and pro-inflammatory cytokines could be potential therapeutic targets in VS

F: female; M: male; *n*: number of cases; NR: not reported; SD: standard deviation; VS: vestibular schwannoma.

### 3.7. Matrix Metalloproteinases

Matrix metalloproteinases (MMPs) are a family of more than 20 zinc endopeptidases that degrade various components of the basal membrane and extracellular matrix [80]. Metalloproteinases and Tissue Inhibitors MetalloProteinases (TIMPs) are secreted by normal cells in various tissues and have a crucial role in physiological activities [81]. An abnormal regulation and secretion of MMPs and TIMPs can lead to pathological conditions (including diabetes mellitus, altered wound healing, renal disorders, neurodegenerative diseases, carcinogenesis) by promoting cell adhesion, proliferation, migration, and apoptosis [82]. In the tumor microenvironment, MMPs have important functions in cell migration and invasion, by degrading basal membrane and extracellular matrix protein components. Moreover, MMPs play roles in other steps of tumor progression, including neo-angiogenesis, modification of signaling pathways, and regulation of the immune response.

Moller et al. [50] investigated MMP-2, MMP-9, and TIMP-1 expressions in non-cystic VSs and their possible correlations with clinic-pathological parameters. They found that all tumors expressed MMP-2, MMP-9, and TIMP-1. A significant correlation was described between the tumor concentration of MMP-9 and the absolute growth rate but not with preoperative tumor size.

Cystic VSs are tumors with a different behavior compared to the solid variant, regarding the growth rate and adhesion to surrounding structures and the facial nerve [51]. It has been hypothesized that MMPs 2 and 14 might play a relevant role in the pathogenesis of cyst formation and peritumoral adhesion. In fact, MMP2 can be found ubiquitously in cyst fluid, and it is localized in the inner luminal surface cells adjacent to the cyst cavity [51]. This suggested that cyst expansion and enlargement could be related to proteolytic activity of MMP-2. Similarly, a higher expression of MMP-14 has been found in the Antoni B compared to the Antoni A area [57].

ADAMs (A disintegrin and metalloproteinase) are a family of trans-membrane and secreted metallo-endopeptidases [83]. One member of this family is ADAM9, which comes into play in cell adhesion and cell signaling and is overexpressed in several tumors [84,85,86]. Breun et al. [25] found that ADAM9 was overexpressed by Schwann cells of VS and its expression was higher in sporadic VSs compared to NF2-associated ones. Moreover, ADAM9 levels significantly correlated with hearing loss.

The cause of sensorineural hearing loss in patients with VS is mechanical compression of the cochlear nerve, as well as the presence of tumor-secreted factors, which contain pro-inflammatory cytokines that can directly cause cochlear damage [87]. Sagers et al. [54] demonstrated this latter mechanism by studying NLRP3 inflammasome expression in a group of VSs. NLRP3 inflammasome is a multi-protein complex made up of apoptosis-associated Speck-like protein with a caspase-recruitment domain, pro-caspase-1, and cryopyrin [88]. In the Sagers et al. [54] investigation, overexpression of multiple key genes associated with the NLRP3 inflammasome was observed in VS compared with control nerves; in addition, two associated proteins (NLRP3, IL-1β) were present in patients with worse hearing loss. These observations confirmed the hypothesis that an important inflammatory response in VS tissue might be associated with cochlear damage and poor hearing levels.

**Table 5:** Studies examining the role of local inflammation proteins in sporadic vestibular schwannomas.

Author	Year	Country	Study Design	Cases (*n*)	Mean Age ± SD (Range)	Sex (M/F)	Tumor Size, Mean ± SD (Range)	Method of Marker Detection	Marker Studied	Key Findings
Breun et al. [25]	2020	Germany	Ex vivo tissue study	30	51 (NR)	12/18	10 small tumors (T3A or smaller) 20 large tumors (T3B of larger)	IHC WB RT-qPCR	ADAM9	- Strong correlation between ADAM9 mRNA and level of hearing impairment - ADAM9 mRNA was overexpressed in the tumor samples compared to healthy vestibular nerves - ADAM9 could be a prognostic marker for VS
Møller et al. [50]	2010	Denmark	Ex vivo tissue study	34	53.61 ± 12.06 (25–75)	14/20	1.686 ± 1.247 cm^3^ (0.11–4.716)	IHC ELISA	MMP-2 MMP-9 TIMP-1	- VS expressed MMP-2, MMP-9 and TIMP-1; MMP-9 correlated with tumor growth rate
Moon et al. [51]	2007	South Korea	Ex vivo tissue study	24	40.5 (16–71)	12/12	43.8 mm (22–60)	Gelatin zymography IHC	MMP-2	- Resection of cystic VS may be complicated by severe adhesion of the capsule to the facial nerve and the large size of the tumor - MMP-2 could be involved in the pathogenesis of cyst formation and aggravate adhesion to the facial nerve
Sagers et al. [54]	2019	USA	Ex vivo tissue study	80	NR	NR	NR	IHC qRT-PCR	NLRP3 (CASP1, PYCARD, IL-18, NLRP3, NAIP, NLRC4, AIM2, IL-1β)	- Overexpression of key components of the NLRP3 inflammasome was preferentially associated with tumors that cause increased hearing loss in VS patients

F: female; M: male; *n*: number of cases; NR: not reported; SD: standard deviation; VS: vestibular schwannoma.

### 3.8. Hormone Receptors

TME is a dynamic network of stromal and immune cells that, under the effect of tumor cells, control biochemical and mechanical signaling via extracellular matrix production and remodeling, formation of vascular networks, and promotion of tumor growth. In other well-known tumors (e.g., breast cancer), hormone receptor-mediated signaling is a key controller of cancer cell proliferation and invasiveness, which occurs both through cell-autonomous means and via cancer cell–stroma crosstalk. In the absence of hormone receptors, a different microenvironment landscape emerges with its own challenges for therapy [89].

Estrogen receptors (ER) may be detected in intracranial tumors as well as VSs [90,91]. VSs are more frequent in females, and an increased growth during pregnancy has been described [92]. In 1981, Kasantikul and Brown [90] first demonstrated estradiol receptors in 8 VSs. Many diverging studies have been published on the contents of sex hormone receptors in VSs. They assumed estrogen and progesterone association with proliferation of VSs [37,90,93,94,95,96]. These studies tried to ascertain a role of sex hormones in considering endocrinological therapy, especially in recurrent and residual cases where complete excision was not feasible. However, the clinical significance of estrogen and progesterone receptors (PR) in VS is still controversial. As hypothesized by Jailswal et al. [36], these discrepancies were probably due to the divergent methodologies that have been used by various research groups, ranging from immunohistochemical methods to molecular techniques. Kasantikul and Brown [90] stated that estrogens might promote tumor growth by inducing vascular endothelium proliferation with a resultant increase in tumor vascularization. Monsell and Wiet [93] investigated 37 cases of VS for estrogen and PR by radioimmunoassay. They found no correlation between ER positivity and the sex of the patient. In a consecutive series of 18 VSs, Klinken et al. [37] found neither estrogen nor PR in clinically relevant quantities.

In 59 cases of VS, Cafer et al. [27] investigated the presence of Ki-67, estrogen and progesterone hormone receptors as well as their clinical correlates. All samples were positive for PR and negative for ER staining. The authors concluded that estrogen is not relevant in VS due to its absence in the tissue samples. Regarding progesterone, since its receptor was expressed in all samples, further studies are needed to evaluate the potential inhibitory effect of anti-progesterone treatment on growth. Labit-Bouvier et al. [41] analyzed 69 cases of VSs and found that 5 were focally positive for PR and none for ER. Curley et al. [96] found no evidence to support the clinical hypothesis that VS might be a hormone-dependent tumor. Dalgorf et al. [97] studied 9 female patients with VS for expression of estrogen, progesterone and VEGF. Their result for estrogen and PR was negative in all cases, while VEGF was positive in 8 out of 9 cases. Jailswal et al. [36] did not find estrogen and PR positivity in any of their 100 VS cases, showing no evidence to support the clinical hypothesis that VS might be a hormone-dependent tumor.

Regarding other hormones, there is a dearth of knowledge about the roles of erythropoietin (EPO) or erythropoietin-receptor (EPO-R) in VS. EPO and EPO-R seem to play important roles in brain’s development and homeostasis [98]. Dillard et al. [32] found that 13 of 14 VS cases showed positive staining for EPO, 9 positive staining for EPO-R. Tumors with high EPO-R levels tended to be larger than those with low or no staining. 

**Table 6:** Studies examining the role of hormone receptors in sporadic vestibular schwannomas.

Author	Year	Country	Study Design	Cases (*n*)	Mean Age ± SD (Range)	Sex (M/F)	Tumor Size, Mean ± SD (Range)	Method of Marker Detection	Marker Studied	Key Findings
Cafer et al. [27]	2008	Turkey	Ex vivo tissue study	59	46.8 (14–75)	27/32	21 small size tumors (<19 mm) 35 medium size tumors (20–39 mm) 3 large size tumors (>40 mm)	IHC	Ki-67 ER PR	- Ki-67 antigen is present in all proliferating cells - Ki-67 had no correlation with the clinical parameters
Dillard et al. [32]	2001	USA	Ex vivo tissue study	14	NR	NR	2.41 ± 1.53 cm (1–6)	IHC	EPO EPO-R	- EPO and EPO-R could play a growth-promoting role in the pathogenesis of VS
Jaiswal et al. [36]	2016	India	Ex vivo tissue study	100	37.5 (12–77)	63/37	NR	IHC	ER PR	- No evidence to support the hypothesis that VS might be a hormone-dependent tumor - No ER or PR could be found
Klinken et al. [37]	1990	Denmark	Ex vivo tissue study	18	52 (26–73)	7/11	NR	IHC	ER PR	- Estrogen and progesterone did not play a role in the growth of VS

ER: estrogen receptor; F: female; M: male; *n*: number of cases; NR: not reported; PR: progesterone receptor; SD: standard deviation; VS: vestibular schwannoma. They concluded that EPO and EPO-R might play a growth-promoting role in the pathogenesis of VSs.

The possibility that VS could be hormone dependent is worthy of investigation because of the success of hormonal manipulation treatments in other areas (e.g., breast and prostatic malignancies). Although some studies identified progesterone and EPO levels in VS, the presence of these hormones in the TME of VS is still controversial.

## 4. Conclusions

The sporadic VS microenvironment is determined by the interplay of several cellular and molecular factors at different levels. A dynamic network of stromal and immune cells interplays under the influence of tumor cells, producing and remodeling extracellular matrix and vascular networks and promoting tumor growth. However, results are sometimes conflicting, which sheds light on how far we are from understanding the complex interactions in the VS microenvironment. Mostly due to the rarity of VS but also to the high costs of molecular-based research, the literature on this topic is based on retrospective ex vivo studies with limited sample size.

Future investigations into the role of TME in VS will further enhance our understanding of the tumor biology, investigating biomarkers predicting tumor growth and recognizing immunological and molecular factors that could be potential therapeutic targets for medical treatment.

## Data Availability

The datasets generated and analyzed during the current study are available on reasonable request.

## Supplementary Materials

The following supporting information can be downloaded at: https://www.mdpi.com/article/10.3390/ijms24076522/s1.
