# Supplementary material for: Tumor Microenvironment in Sporadic Vestibular Schwannoma: A Systematic, Narrative Review
Source: Int J Mol Sci. 2023 Mar 30;24(7):6522. doi: 10.3390/ijms24076522 (PMC10094882; doi:10.3390/ijms24076522)
Supplement: Supplementary file 1 [file ijms-24-06522-s001.zip › ijms-2258141-supplementary.pdf]

SEARCH QUERIES

PUBMED

("neuroma, acoustic"[MeSH Terms] OR "vestibular schwannoma"[All Fields] OR "acoustic neuroma"[All Fields]) AND ("Cellular Microenvironment"[MeSH Terms] OR "Tumor Microenvironment"[MeSH Terms] OR "Immunohistochemistry"[MeSH Terms] OR "Intercellular Signaling Peptides and Proteins"[MeSH Terms] OR ("microenvironment"[All Fields] OR "microenvironment s"[All Fields] OR "microenvironments"[All Fields]) OR "immunohistochemical profile"[All Fields] OR ("immunohistochemistries"[All Fields] OR "Immunohistochemistry"[MeSH Terms] OR "Immunohistochemistry"[All Fields]) OR "growth factor"[All Fields]).

SCOPUS

((TITLE-ABS-KEY (microenvironment) OR TITLE-ABS-KEY ("immunohistochemical profile") OR TITLE-ABS-KEY ("Immunohistochemistry") OR TITLE-ABS-KEY ("growth factor"))) AND ((TITLE-ABS-KEY ("vestibular schwannoma") OR TITLE-ABS-KEY ("acoustic neuroma"))) AND (LIMIT-TO (OA , "all")) AND (LIMIT-TO (SUBJAREA , "MEDI")).

WEB OF SCIENCE

((TITLE-ABS-KEY (microenvironment) OR TITLE-ABS-KEY ("immunohistochemical profile") OR TITLE-ABS-KEY ("Immunohistochemistry") OR TITLE-ABS-KEY ("growth factor"))) AND ((TITLE-ABS-KEY ("vestibular schwannoma") OR TITLE-ABS-KEY ("acoustic neuroma"))) AND (LIMIT-TO (OA , "all")) AND (LIMIT-TO (SUBJAREA , "MEDI")).

	Author	Year	Quality assessment
ANGIOGENESIS	Brieger et al. [26]	2003	Good
	Cayè-Thomasen et al. [28]	2005	Fair
	Koutsimpelas et al. [38]	2007	Good
	Koutsimpelas et al. [39]	2012	Good
	Marioni et al. [46]	2019	Good
	Matsunaga et al. [48]	1996	Fair
	Xia et al. [57]	2020	Poor
IMMUNO INFILTRATE	Amit et al. [23]	2022	Fair
	De Vries et al. [29]	2012	Good
	De Vries et al. [30]	2019	Good
	Gonçalves et al. [34]	2021	Fair
	Labit-Bouvier et al. [41]	2000	Fair
	Leisz et al. [43]	2022	Good
	Lewis et al. [44]	2021	Fair
	Perry et al. [53]	2020	Fair
MOLECULAR REGULATORS	Ahmad et al. [21]	2009	Good
	Breun et al. [24]	2018	Fair
	Jabbour et al. [35]	2016	Fair
	Lassaletta et al. [42]	2011	Good
	Martini et al. [47]	2017	Fair
	Mawrin et al. [49]	2002	Fair
	Neff et al. [52]	2006	Poor
	Seol et al. [55]	2005	Poor
GROWTH FACTORS	Altuna et al. [22]	2011	Good
	Diensthuber et al. [31]	2004	Good
	Dilwali et al. [33]	2013	Poor
	Kramer et al. [40]	2010	Poor
	Löttrich et al. [45]	2007	Poor
	Taurone et al. [56]	2015	Fair

LOCAL INFLAMMATION PROTEINS	Breun et al. [25]	2020	Fair
	Møller et al. [50]	2010	Good
	Moon et al. [51]	2007	Fair
	Sagers et al. [54]	2019	Poor
HORMONE RECEPTORS	Cafer et al. [27]	2008	Fair
	Dillard et al. [32]	2001	Poor
	Jaiswal et al. [36]	2016	Fair
	Klinken et al. [37]	1990	Poor

Table S1. Quality assessment of the included studies.
